# Supplementary material for: Perceived built environment, health-related quality of life and health care utilization
Source: PLoS One. 2021 May 6;16(5):e0251251. doi: 10.1371/journal.pone.0251251 (PMC8101743; doi:10.1371/journal.pone.0251251)
Supplement: S1 Appendix — (DOCX) [file pone.0251251.s001.docx]

Supplementary information:

Table A1 Questions on the perceived built and social environment

| Satisfaction with Apartment / Neighborhood  0-4 rating scale | How satisfied are you with… |
| --- | --- |
|  | Your apartment |
|  | Your immediate living environment |
|  | Your residential community |
|  | Your neighborhood |
| Proximity  1=1-5min  2=6-10min  3=11-20min  4=21-30min  5=>30min | How long does it take to walk from your house to the nearest shops, public facilities or recreation areas? |
|  | Local Shops |
|  | Supermarkets |
|  | Local facilities |
|  | Restaurants, Café, Bars |
|  | Fast-Food-Restaurant |
|  | Public Transportation |
|  | Sports facilities |
|  | Recreation areas |
|  | Quite/Green Places |
| Noise annoyance  0-10 rating scale | How much are you annoyed by transportation noise in your home when the windows are open? |
| Living alone / with partner | As the marital status does not necessarily correspond to the way of life at the present time, we ask you to indicate whether you live alone or in partnership |
| Occupational status | What best describes your current job situation? |
|  | Full-time working (>80%) |
|  | Full-time Housewife/man (<80%) |
|  | Part-time working (<80%) |
|  | Hourly or irregularly employed |
|  | Unemployed |
|  | Not in employment because of education or longer vacation |
|  | Not employed because sick or disabled |
|  | Retired |
|  | Retired but still occupationally active |
| Social engagement  0-6 rating scale | The following list contains a number of groups or clubs that are visited during leisure time, as well as activities that can be carried out during leisure time. How often do you actively participate in the listed activities? |
|  | Sports Clubs & Fitness Centers |
|  | Hobby clubs, allotment garden clubs, shooting clubs, choirs, music and theater clubs |
|  | Professional organizations, professional associations, unions |
|  | Parish, religious associations |
|  | Political groups/parties |
|  | Self-help groups |
|  | Visits to cultural events, museums |
|  | Hiking groups, card game groups |
|  | Family/Friends/Neighbours Meetings |
|  | Voluntary work |


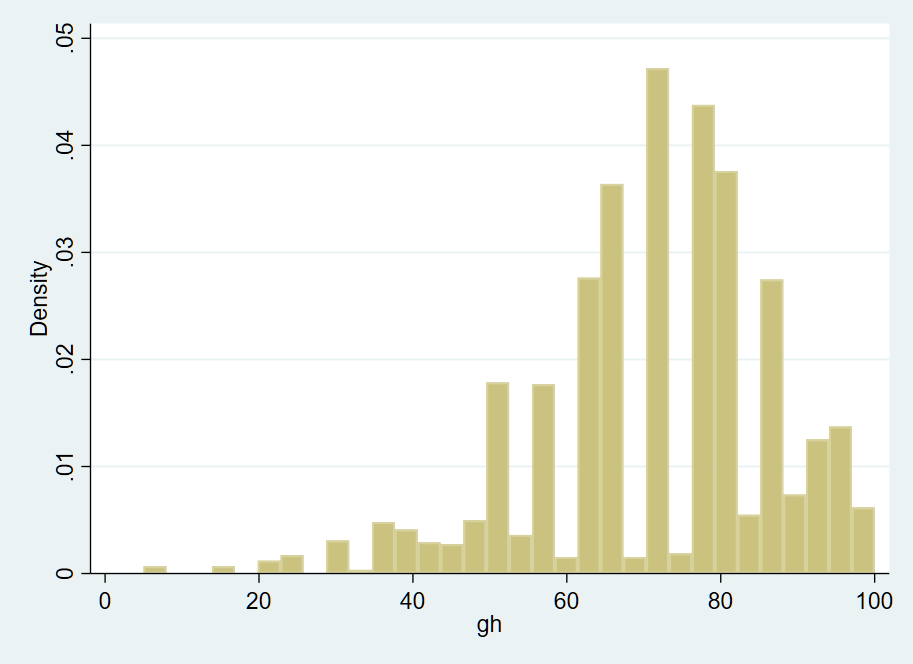

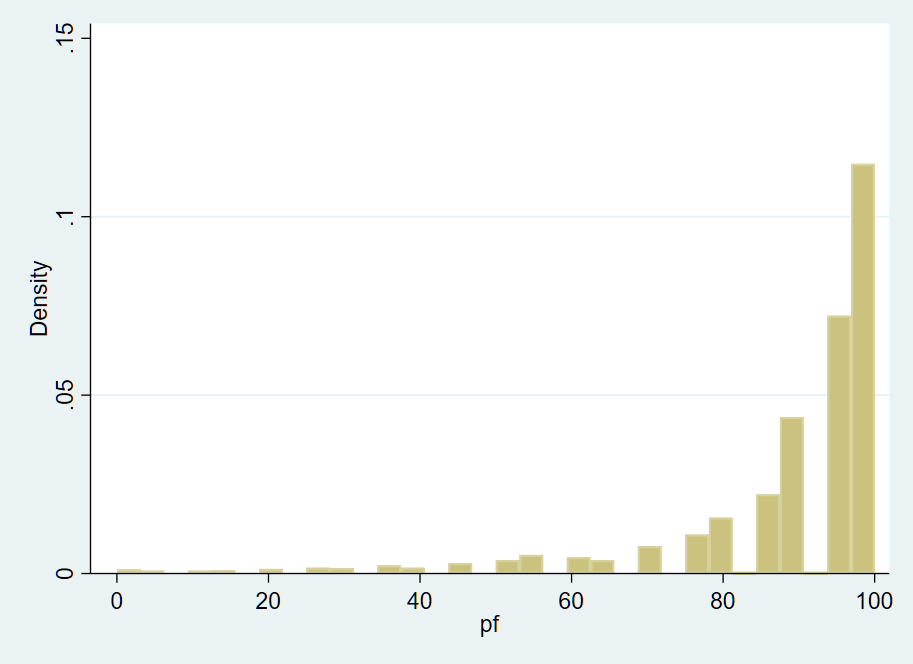

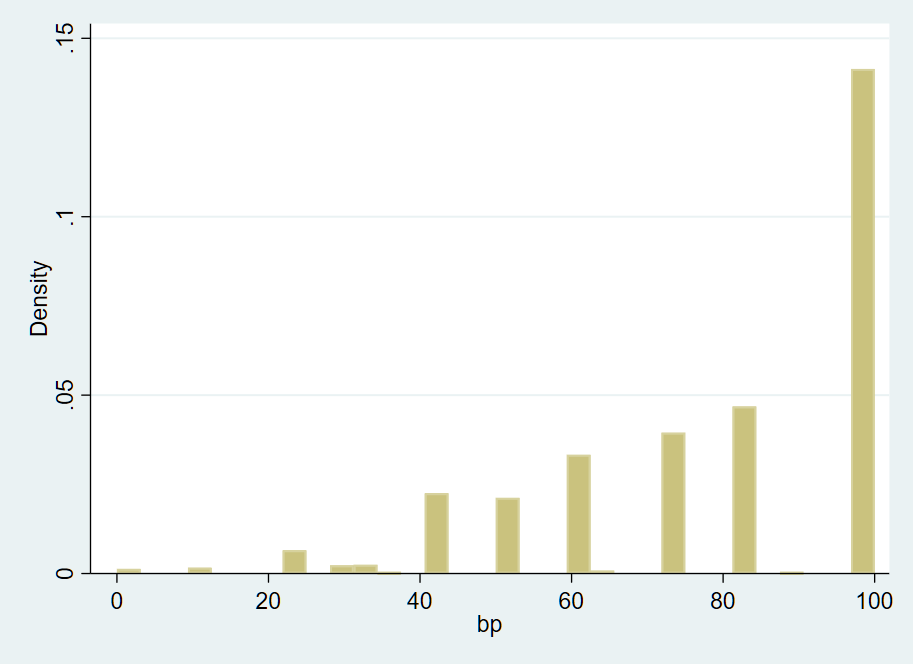

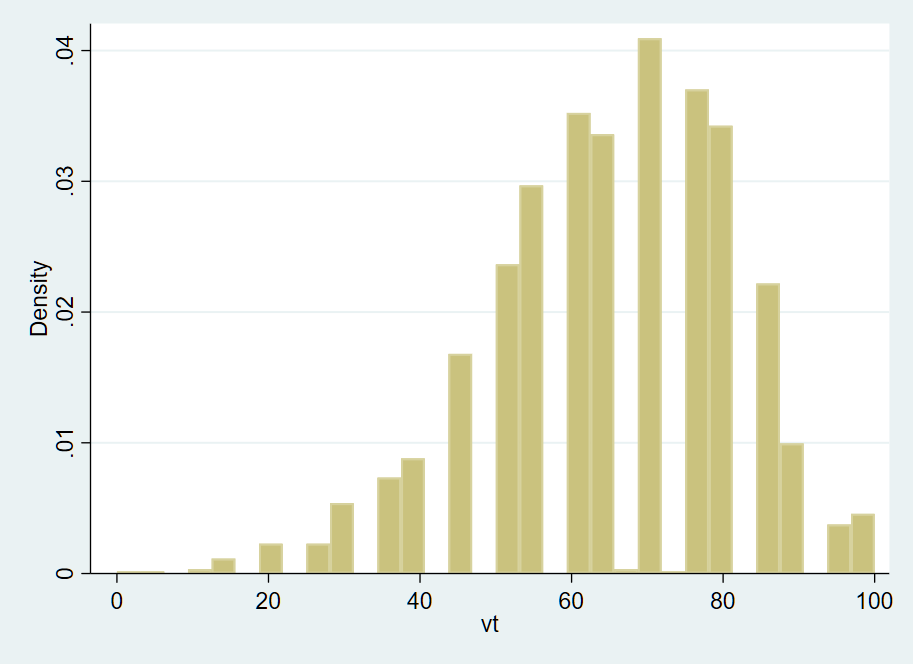

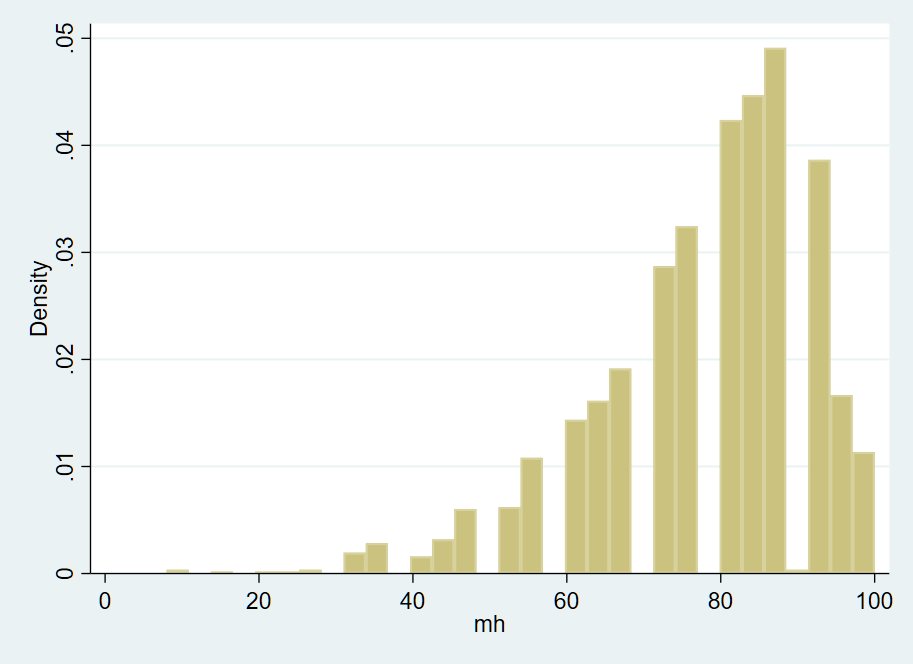


Figure A1. Histograms of SF-36 derived health-related quality of life measures (GH, General Health; PF, Physical functioning; BP, Bodily Pain; VT, Vitality; MH, Mental Health)

Table A2. Spearman’s rank correlation of perceived built and social environment variables

|  | Satisfaction neighborhood | Proximity Social Places | Proximity Public Transportation | Proximity Sports Facilities | Proximity Quite/Green Places | Noise annoyance | Living alone vs. with partner | Social engagement |
| --- | --- | --- | --- | --- | --- | --- | --- | --- |
| Satisfaction neighborhood | 1.0000 |  |  |  |  |  |  |  |
| Proximity Social Places | -0.0815* | 1.0000 |  |  |  |  |  |  |
| Proximity Public Transportation | -0.0454* | 0.2561* | 1.0000 |  |  |  |  |  |
| Proximity Sports Facilities | 0.0135 | 0.5224* | 0.1690* | 1.0000 |  |  |  |  |
| Proximity Quite/Green Places | 0.1645* | -0.0254 | 0.0851* | 0.1274* | 1.0000 |  |  |  |
| Noise annoyance | -0.1676* | 0.0162 | -0.0264 | -0.0261 | -0.0047 | 1.0000 |  |  |
| Living alone vs. with partner | 0.0788* | -0.1007* | -0.0406 | -0.0053 | 0.0618* | -0.0401 | 1.0000 |  |
| Social engagement | 0.0574* | -0.0043 | 0.0012 | 0.0520* | 0.0389 | -0.0107 | -0.0079 | 1.0000 |
